# Histopathological features of asymmetric lacrimal gland enlargement in patients with thyroid eye disease

**DOI:** 10.1186/s13044-023-00174-4

**Published:** 2023-08-14

**Authors:** Pav Gounder, Huw Oliphant, Valerie Juniat, Michael Koenig, Dinesh Selva, Saul N. Rajak

**Affiliations:** 1 The Sussex Eye Hospital, Brighton and Sussex University Hospitals NHS Trust, Eastern Road, Brighton, BN2 5BF UK; 2 Centre of Ophthalmology and Visual Science, The University of Western Australia, Perth, WA Australia; 3 Brighton and Sussex Medical School, Falmer, Brighton, BN1 9PX UK; 4 South Australian Institute of Ophthalmology, Royal Adelaide Hospital, Adelaide, South Australia Australia; 5 Department of Cellular Pathology, University Hospitals Sussex NHS Foundation Trust, Brighton, UK

**Keywords:** Lacrimal gland enlargement, Thyroid eye disease, Histopathology, Thyroid ophthalmopathy, Graves’ disease

## Abstract

**Purpose:**

Lacrimal gland enlargement can be a feature of thyroid eye disease (TED). Unilateral or asymmetric lacrimal gland enlargement is poorly described and may impede diagnosis. We present the histological and clinical findings of four patients with asymmetric lacrimal gland enlargement.

**Methods:**

A retrospective case note review was performed for patients over two tertiary orbital clinics (Royal Adelaide Hospital, South Australia and the Sussex Eye Hospital, Brighton, United Kingdom) presenting with an asymmetrical lacrimal gland enlargement with a background of TED that underwent biopsy to exclude alternate diagnoses. Baseline data was collected for each patient and histopathological images and reports were reviewed.

**Results:**

All four patients were hyperthyroid at time of lacrimal gland biopsy. Biopsy demonstrated nonspecific, lymphoid aggregates, typically of B cell type, with no diagnostic findings to support lymphocyte clonality or IgG4-related disease. One biopsy specimen demonstrated evidence of some fibrosis.

**Conclusion:**

Asymmetrical lacrimal gland enlargement can occur as part of the TED spectrum but may require biopsy to exclude alternate pathology. Histology demonstrates a non-specific lymphocytic infiltrate.

## Introduction

Thyroid eye disease (TED) is the commonest cause of orbital inflammation in adults. It can result in expansion and fibrosis of the extraocular muscles and orbital fat [1]. Radiological evidence of symmetrical lacrimal gland enlargement secondary to TED has been demonstrated, although asymmetric lacrimal enlargement is atypical and poorly described in the literature [2–5].

The purpose of this study is to report on the histopathological findings of lacrimal gland biopsies in patients with asymmetric lacrimal gland enlargement.

## Methods

A retrospective review of clinical case notes was performed for all patients presenting to two units (Royal Adelaide Hospital, South Australia and the Sussex Eye Hospital, United Kingdom) between the years 2013 to 2020. Inclusion criteria included patients with known thyroid dysfunction, upper eyelid retraction and/or lid lag in keeping with thyroid eye disease, clinical and/or radiological findings of lacrimal gland enlargement, magnetic resonance imaging (MRI) of the orbits and subsequent lacrimal gland biopsy and serological investigations (eg serum immunoglobulin subclasses) to exclude concurrent disease.

Baseline data for each patient was collected including age, sex, ocular comorbidity, laterality, ophthalmic findings, and histopathological findings. Clinical Activity Score (CAS) was obtained for the patient’s initial presentation. The CAS evaluates inflammatory signs and symptoms that are often characteristic of active TED.

Ethical approval for this study was not required as per the Human Research Authority (HRA) online decision-making tool. Informed consent was provided by each patient for the procedure and use of clinical information.

## Results

Four patients were identified through case note review (Table 1). The mean age of the patients at time of biopsy was 58 years and 9 months (Range of 42–68). Three patients were female.

**Table 1:** Summary of cases with asymmetrical lacrimal gland swelling

Case	Age	Gender	Medical History	Presentation	Thyroid status	Clinical Activity Score	MRI Findings	Lacrimal Gland Biopsy	Histopathology
1	62	M	Asthma	Left upper lid retraction 4mm left proptosis No treatment prior to biopsy	Hyperthyroid	2/7	Asymmetrical left lacrimal gland enlargement	Left lacrimal gland biopsy 27 days after presentation	Preservation of the architecture within the lacrimal gland. There are occasional focal areas of lymphocytes and occasional plasma cells within the lobules
2	42	F	HIV	5mm left proptosis Limitation of abduction OS Limitation of elevation OS No treatment prior to biopsy	Hyperthyroid	1/7	Asymmetrical eft lacrimal gland enlargement	Left lacrimal gland biopsy 13 days after presentation	Extensive infiltration of small mature lymphocytes, surround and infiltrate lacrimal ducts. Occasional plasma cells. Expanded B cell population, no evidence of monoclonality. Extensive positivity for IgG but not IgG4
3	63	F	Morbid obesity Type 2 Diabetes Mellitus Hypertension Polycystic Ovarian Syndrome	Right upper lid retraction Oedema of right upper lid Right lid lag Right upper lid subconjunctival triamcinolone injection at time of presentation	Hyperthyroid	1/7	Asymmetrical right lacrimal gland enlargement	Right lacrimal gland biopsy 76 days after presentation	Small lymphoid population without evidence of fibrosis
4	68	F	Nil	Left upper lid retraction Left lid lag No treatment prior to biopsy	Hyperthyroid	2/7	Asymmetrical left lacrimal gland enlargement	Left lacrimal gland biopsy 20 days after presentation	Small patchy and predominantly lymphoid infiltrates. Presence of fibrosis. Mixed B cell population. CD20 + B Cells and CD3 + ve T Cells

Three of the four patients presented with upper lid retraction. All four patients had inactive disease. Other common presentation features included lid lag and proptosis. MRI had been requested as routine baseline imaging of the orbital contents.

**Fig. 1:**
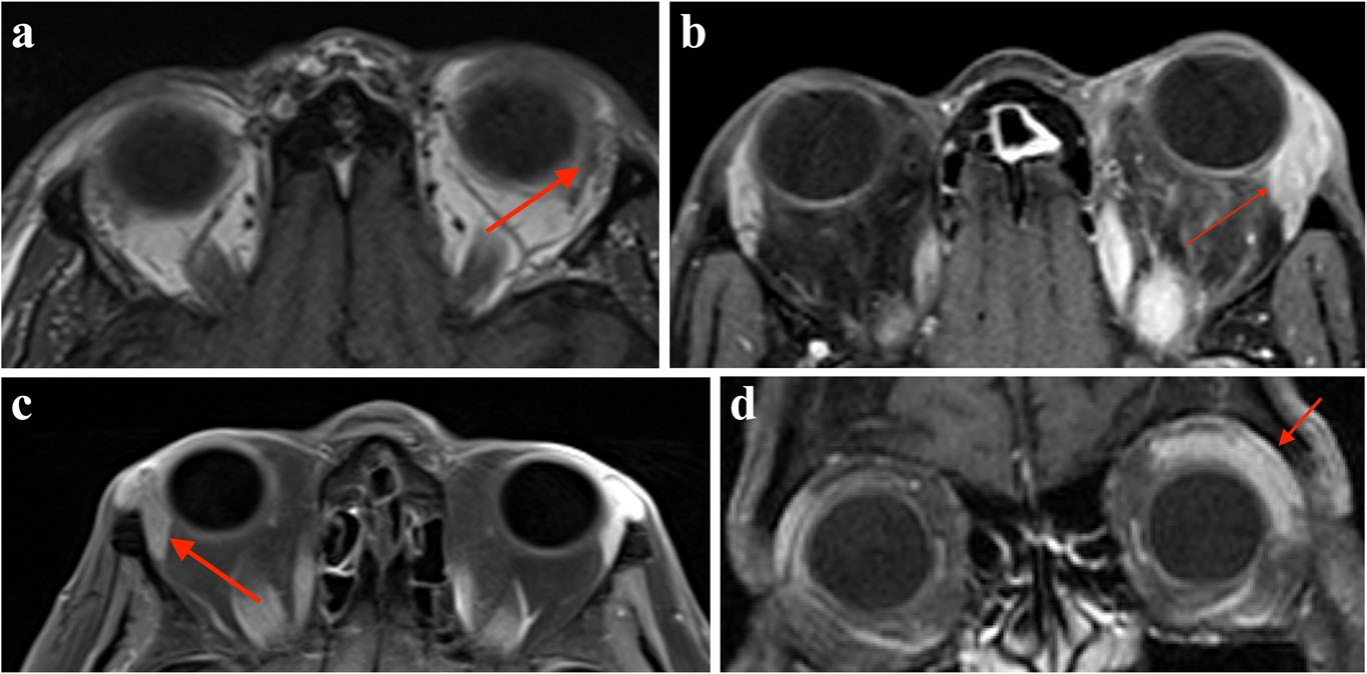
**a** MRI T1 image (axial view) of Case 1 demonstrating left proptosis and a marginally larger left lacrimal gland. **b** MRI T1 Fat suppressed post contrast image (axial view) of Case 2 demonstrating left proptosis and left lacrimal gland enlargement. **c** MRI T1 Fat suppressed post contrast image (axial view) of Case 3 demonstrating right lacrimal gland enlargement. 1d MRI T1 Fat suppressed post contrast image (coronal view) of Case 4 demonstrating left lacrimal gland enlargement. Arrows are included in images indicating the asymmetrical lacrimal gland enlargement

All patients were biochemically hyperthyroid and demonstrated asymmetrical lacrimal gland enlargement on MRI (Fig. 1 a, b, c, d).

**Fig. 2:**
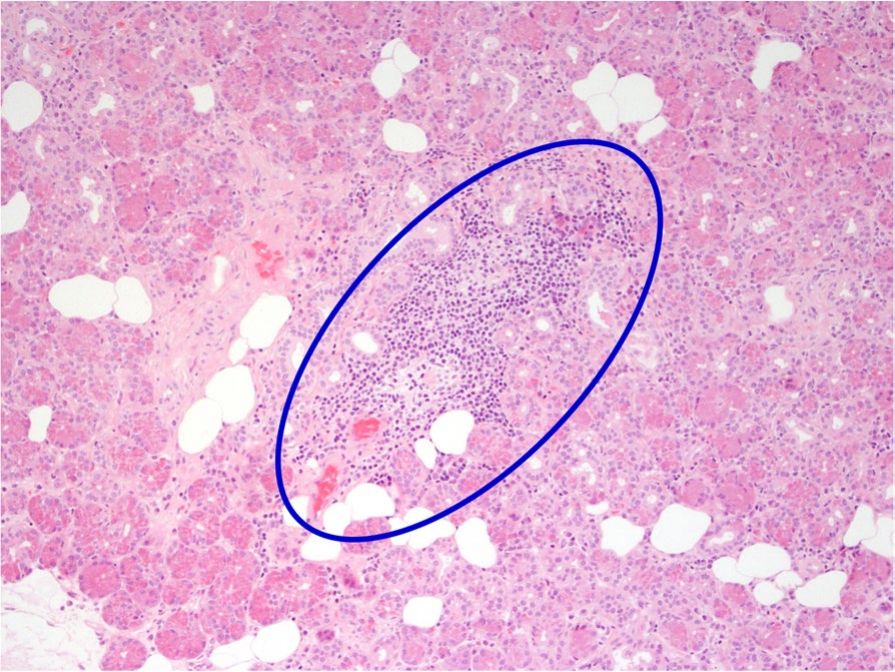
Case 1 Lacrimal gland serous tissue with small infiltrates of lymphocytes (circled blue) and sparse plasma cells, H&E staining

**Fig. 3:**
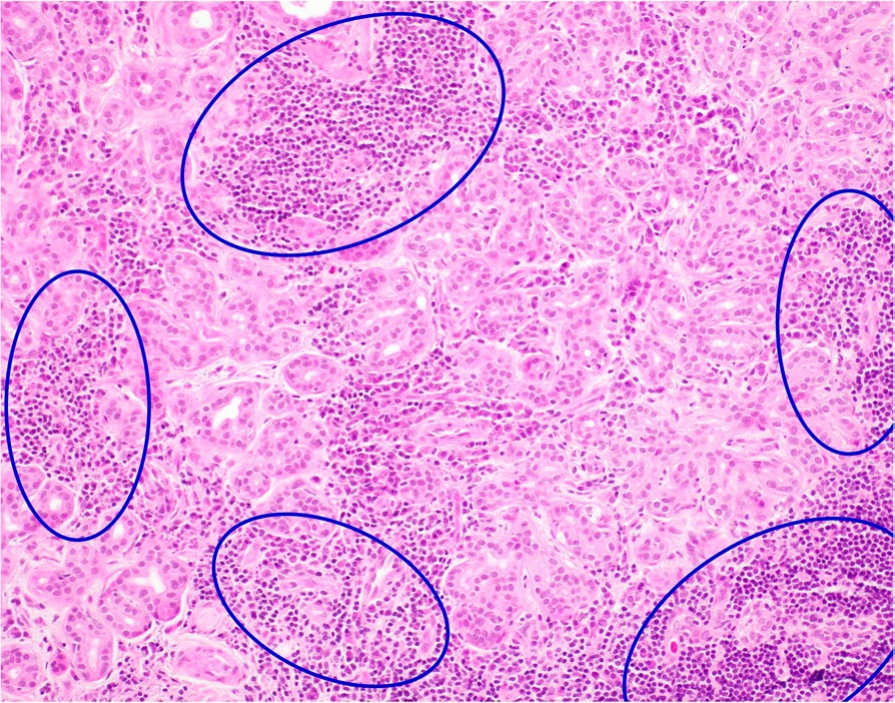
Case 2 lacrimal gland with largely lymphocytic infiltrate (circled blue) with some plasma cells, H&E staining

**Fig. 4:**
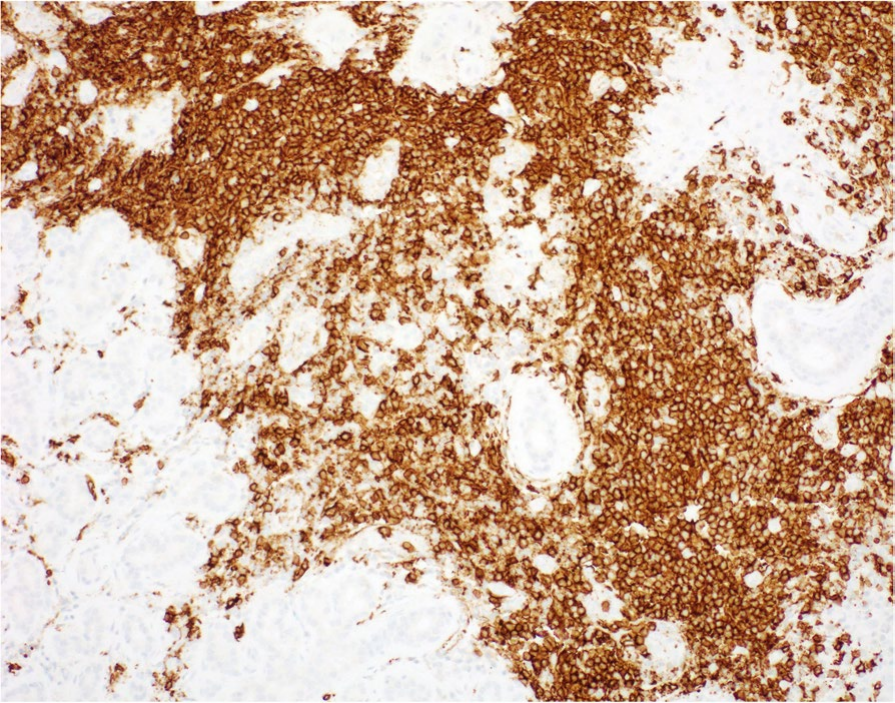
Case 2 lacrimal gland histopathology highlighting IgG positive B cells in brown (IgG4 negative)

**Fig. 5:**
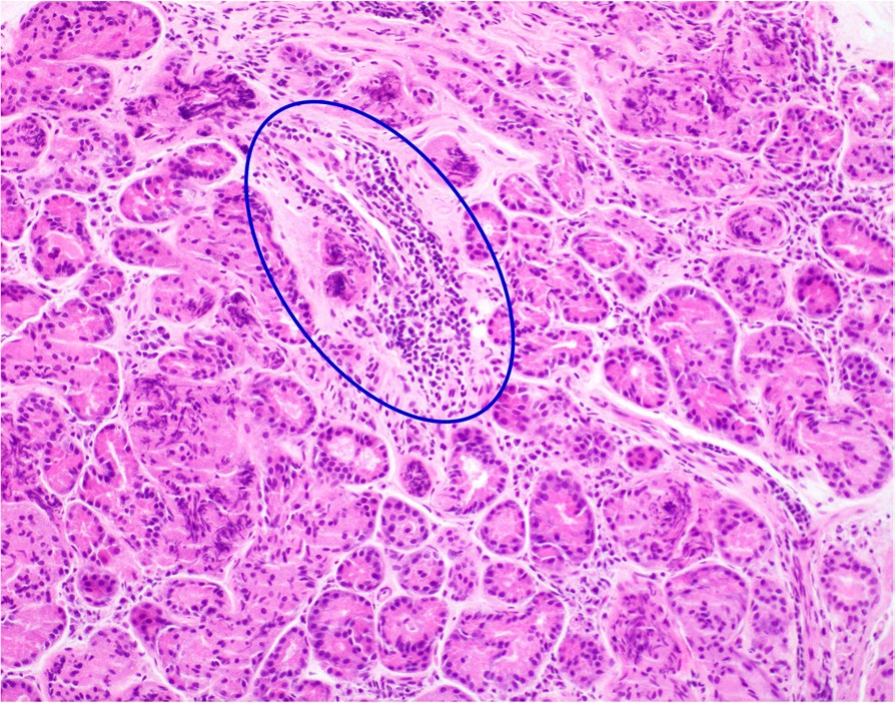
Case 4 lacrimal gland histopathology with patchy infiltration by lymphocytes (circled blue) and very sparse fibrosis, H&E staining

Histopathological analysis of lacrimal gland biopsy specimens showed nonspecific chronic inflammation composed largely of B lymphocytes in all four cases (Figs. 2, 3, 4). Only one case demonstrated some mild fibrosis in the biopsy sample (Fig. 5). Features suggestive of IgG4-related disease were absent and clonality studies negative in all cases.

## Discussion

Lacrimal gland enlargement is a common finding in TED and particularly in the active stage of the disease [4, 6, 7]. Asymmetric lacrimal gland enlargement on the background of thyroid eye disease is atypical and warrants further investigation given the possibility of malignancy and other inflammatory diseases. Ishikawa et al. presented a series of 16 patients with TED and asymmetrical lacrimal enlargement. Biopsies on 9 patients revealed disease entities other than thyroid eye disease such as IgG4-related disease, sarcoidosis and MALT lymphoma [3]. The potential for malignant and severe inflammatory disease means the threshold for biopsy should be low when faced with asymmetrical lacrimal gland enlargement.

Radiological findings may assist the decision-making regarding biopsy of the lacrimal gland. Epithelial neoplasms predominantly involve the orbital lobe whilst inflammatory and lymphoproliferative lesions tend to involve both the orbital and palpebral lobes [8]. Lymphoproliferative disease may also cause bony scalloping, which is generally not seen with inflammatory lesions [8]. Diffusion-weighted imaging (DWI) can be informative: typically the apparent diffusion coefficient (ADC) of the lacrimal gland is low in lymphoma but may be high in TED when compared to healthy controls [9, 10]. The ADC of the lacrimal gland also has a high predictive value to determine active compared to inactive TED. Lacrimal gland features on MRI may assist in staging TED. The signal intensity ratio of the lacrimal gland to the ipsilateral temporal muscle on fat suppressed T2 weighted MRI imaging has shown promise in staging active TED [11]. Patients with active TED demonstrate a higher signal intensity ratio (SIR) of the lacrimal gland to the ipsilateral temporal muscle compared against patients with inactive TED. Clinical features and a careful history may also aid the decision on whether to biopsy the lacrimal gland. Lacrimal gland prolapse is also more likely in active thyroid eye disease [6, 12]. However, the presence of a prolapsed lacrimal gland may make clinical judgement of an enlarged lacrimal gland more difficult clinically and therefore radiological measurements are required.

Previously reported lacrimal gland histopathological findings in TED have also shown nonspecific inflammatory cell infiltration with and without fibrosis [3, 13, 14]. Of the patients with nonspecific dacryoadenitis in the Ishikawa et al. series, inflammatory cells were present with a small amount of fibrotic change in all seven patients and two patients demonstrated germinal centres [3]. Yahalomi et al. described a case of asymmetric lacrimal gland enlargement in a patient with thyroid eye disease with histopathology demonstrating periacinar lymphocytic infiltration [13]. Khu et al. also presented a case report of asymmetric lacrimal gland enlargement in the setting of thyroid eye disease with biopsy showing a mild chronic inflammatory infiltrate composed of plasma cells [14]. The normal lacrimal gland becomes increasingly fibrotic with age [15]. The oldest patient at time of biopsy (68) in our series was the only biopsy specimen to demonstrate fibrosis; it is uncertain if this derives from age or TED chronicity. The residual inflammation present in the biopsies may be an indicator of grumbling inflammation despite all four patients being clinically inactive. Although not present in our series, germinal centres have previously been described in TED-related lacrimal gland enlargement [3]. This finding is also consistent with follicular type idiopathic orbital inflammation (IOI) [16, 17].

These nonspecific histological changes, to some extent, diagnoses thyroid eye disease by exclusion, given the lack of a specific histopathological marker, although there remains the possibility of other concurrent, non-specific, diseases such as idiopathic dacryoadenitis that tend to present unilaterally.

All four patients in the present study were hyperthyroid which contrasts with previous studies which have shown asymmetric TED clinical presentations predominantly in euthyroid or hypothyroid disease states [3, 14, 18, 19]. Fourteen of the sixteen patients analysed in the Ishikawa et al. series on asymmetrical lacrimal gland enlargement were euthyroid [3]. In Eckstein et al.’s study comparing TED clinical symptoms in hyperthyroid patients versus hypothyroid and euthyroid patients, the authors postulated that monosymptomatic and asymmetrical manifestations in euthyroid patients may lead to diagnostic difficulty, increasing the use of imaging by clinicians to confirm diagnosis. Sub-clinical enlargement of the lacrimal gland may therefore be revealed more often in euthyroid and hypothyroid patients through imaging [18].

There are several limitations to this study. Firstly, the retrospective and non-comparative design limit inferences that can be drawn. Secondly, a standard set of immunohistochemical testing was not undertaken due to the retrospective and multi-centre nature of the study. Finally, we have included a small number of patients, although this may reflect the relatively uncommon nature of TED-related, asymmetric lacrimal gland enlargement.

## Conclusion

This study confirms that those patients with lacrimal gland enlargement presumed secondary to TED typically show nonspecific chronic inflammatory changes on histopathological analysis. Lacrimal gland biopsy is prudent given concurrent malignant or inflammatory disease is possible with asymmetrical enlargement although radiological imaging modalities of the lacrimal gland show promise in diagnosing active TED.

## Data Availability

All data is made available in the manuscript.
